# ASSOCIATION BETWEEN GUT MICROBIOTA, DIET, AND COGNITION IN ELDERLY PATIENTS WITH MILD COGNITIVE IMPAIRMENT

**DOI:** 10.1093/geroni/igae098.2967

**Published:** 2024-12-31

**Authors:** Lili Chen, Zhaoyi Hou, Jinxiu Liu, Xiuli Li, Yuping Zhang, Shuhui Yang, Peiling Zhang

**Affiliations:** Fujian Provincial Hospital, Fuzhou, Fujian, China (People’s Republic); Fujian Medical University, Fuzhou, Fujian, China (People’s Republic); Fujian Medical University, Fuzhou, Fujian, China (People’s Republic); Fujian Medical University, Fuzhou, Fujian, China (People’s Republic); Fujian Medical University, Fuzhou, Fujian, China (People’s Republic); Fujian Medical University, Fuzhou, Fujian, China (People’s Republic); Fujian Medical University, Fuzhou, Fujian, China (People’s Republic)

## Abstract

**Objectives:**

To compare the gut microbiota in individuals with mild cognitive impairment (MCI) and normal controls (NCs) and explore the association between dietary intake and various cognitive domains across identified enterotypes, providing a basis for dietary interventions based on the Microbiota-Gut-Brain Axis.

**Methods:**

We analyzed fecal samples and 16S ribosomal RNA sequences for microbiota among 100 participants (NCs: 50, MCI: 50). Cognitive function was evaluated using the Montreal Cognitive Assessment (MoCA), Mini-Mental State Examination (MMSE), and Auditory Verbal Learning Test (AVLT). A semi-quantitative food frequency questionnaire was used to assess participants’ dietary intake, and differences in dietary habits and cognitive function among distinct enterotypes were analyzed.

**Results:**

Participants with MCI exhibited lower bacterial richness. Linear Discriminant Analysis of Effect Sizes (LEfSe-LDA) revealed different major microbial genera between the groups, and a random forest model based on dominant bacterial genera could distinguish between MCI and NCs (AUC=0.743, 95%CI:0.646-0.840). Cluster analysis identified two enterotypes: Escherichia-Shigella (enterotype E) and Bacteroides (enterotype B). When grouped by enterotypes, similar dietary intake was associated with different cognitive domains: notably, egg intake positively correlated with MOCA and AVLT scores for enterotype E, but not for enterotype B. Conclusions: Significant differences exist in the gut microbiota of individuals with MCI compared to those with normal cognition, with dominant genera showing good predictive performance. The impact of different enterotypes on the relationship between dietary intake and cognitive function should be considered when making dietary recommendations in clinical practice.

